# Modeling the Effects of Cell Cycle M-phase Transcriptional Inhibition on Circadian Oscillation

**DOI:** 10.1371/journal.pcbi.1000019

**Published:** 2008-03-28

**Authors:** Bin Kang, Yuan-Yuan Li, Xiao Chang, Lei Liu, Yi-Xue Li

**Affiliations:** 1 Bioinformatics Center, Shanghai Institutes for Biological Sciences, Chinese Academy of Sciences, Shanghai, China; 2 Shanghai Center for Bioinformation Technology, Shanghai, China; University of Washington, United States of America

## Abstract

Circadian clocks are endogenous time-keeping systems that temporally organize biological processes. Gating of cell cycle events by a circadian clock is a universal observation that is currently considered a mechanism serving to protect DNA from diurnal exposure to ultraviolet radiation or other mutagens. In this study, we put forward another possibility: that such gating helps to insulate the circadian clock from perturbations induced by transcriptional inhibition during the M phase of the cell cycle. We introduced a periodic pulse of transcriptional inhibition into a previously published mammalian circadian model and simulated the behavior of the modified model under both constant darkness and light–dark cycle conditions. The simulation results under constant darkness indicated that periodic transcriptional inhibition could entrain/lock the circadian clock just as a light–dark cycle does. At equilibrium states, a transcriptional inhibition pulse of certain periods was always locked close to certain circadian phases where inhibition on Per and Bmal1 mRNA synthesis was most balanced. In a light–dark cycle condition, inhibitions imposed at different parts of a circadian period induced different degrees of perturbation to the circadian clock. When imposed at the middle- or late-night phase, the transcriptional inhibition cycle induced the least perturbations to the circadian clock. The late-night time window of least perturbation overlapped with the experimentally observed time window, where mitosis is most frequent. This supports our hypothesis that the circadian clock gates the cell cycle M phase to certain circadian phases to minimize perturbations induced by the latter. This study reveals the hidden effects of the cell division cycle on the circadian clock and, together with the current picture of genome stability maintenance by circadian gating of cell cycle, provides a more comprehensive understanding of the phenomenon of circading gating of cell cycle.

## Author Summary

Circadian clock and cell cycle are two important biological processes that are essential for nearly all eukaryotes. The circadian clock governs day and night 24 h periodic molecular processes and physiological behaviors, while cell cycle controls cell division process. It has been widely observed that cell division does not occur randomly across day and night, but instead is normally confined to specific times during day and night. These observations suggest that cell cycle events are gated by the circadian clock. Regarding the biological benefit and rationale for this intriguing gating phenomena, it has been postulated that circadian gating helps to maintain genome stability by confining radiation-sensitive cell cycle phases to night. Bearing in mind the facts that global transcriptional inhibition occurs at cell division and transcriptional inhibition shifts circadian phases and periods, we postulate that confining cell division to specific circadian times benefits the circadian clock by removing or minimizing the side effects of cell division on the circadian clock. Our results based on computational simulation in this study show that periodic transcriptional inhibition can perturb the circadian clock by altering circadian phases and periods, and the magnitude of the perturbation is clearly circadian phase dependent. Specifically, transcriptional inhibition initiated at certain circadian phases induced minimal perturbation to the circadian clock. These results provide support for our postulation. Our postulation and results point to the importance of the effect of cell division on the circadian clock in the interaction between circadian and cell cycle and suggest that it should be considered together with other factors in the exploitation of circadian cell cycle interaction, especially the phenomena of circadian gating of cell cycle.

## Introduction

For organisms living on the surface of the earth or in shallower aquatic biotopes, the ability to adjust their metabolic processes and behaviors according to a 24-hour periodicity, and the synchronization of their internal molecular processes may provide important evolutionary advantages. Circadian clocks are endogenous time-keeping devices that are responsible for the ≈24-hour biochemical rhythm of almost all organisms ranging from simple single cellular prokaryotes to complex multi-cellular eukaryotes. Circadian clocks coordinate synchronization between internal biological processes and between environmental cues and internal biological processes.

An endogenous circadian clock consists of single or multiple autoregulatory oscillator(s) composed of interconnected transcriptional feedback loops [1]–[4]. These molecular feedback loops contain positive and negative elements. Positive elements activate transcription of the negative elements, while negative elements inhibit the positive elements. This regulatory regime between positive and negative elements causes oscillatory fluctuation of the concentrations of both components. Recent years have seen great advances in deciphering the molecular components and concomitant regulatory logic of circadian controlling systems in at least five model systems: the cyanobacterium Synechococcus elongates, the filamentous fungus Neurospora crassa, the fruitfly Drosophila melanogaster, plant and mammals [5]. One important feature of circadian clock is that it is flexible in response to environmental and physiological changes and can be entrained or reset by many environmental factors like light, food cues and many other physiological chemical factors [6]–[9]. Chemicals with transcriptional inhibition activity has also been reported being able to entrain the circadian clock [10]. With this flexibility, circadian clocks can easily adapt to environmental conditions and reconcile and coordinate various physiological processes.

The cell cycle is another fundamental clock-like periodic biological process for which interesting molecular details have been elucidated. At the molecular level, a similar regulatory scenario to the circadian clock is observed, with transcriptional and translational feedback loops underlying the cell cycle engine mechanism. The phenomena of coupling between cell cycle and circadian cycle were observed and investigated over 40 years ago [11],[12]. In 1964, Edmunds *et.al*. found that the autotrophic Euglena gracilis Klebs, grown on defined medium with a regime of 14 hours of light and 10 hours of darkness, double their cell number every 24 hours, dividing synchronously during the dark period [13]. This observation was subsequently further confirmed by Edmunds' group [12],[14],[15]. Such circadian phase specific distribution of cell cycle phases of DNA synthesis or mitosis was also observed in mammals both *in vivo* and *in vitro* [16] and even in tumor cells [17]. In the last few decades, this phenomenon was also observed in many other organisms [18],[19]. These observations were all interpreted as gating of specific events of cell division by a circadian clock [11], [20]–[22].

This prompts two questions. Why is there widespread gating of the cell cycle by a circadian clock mechanism in most organisms? And is there any reciprocal “gating” effect of the cell cycle on the circadian clock? As yet, there is no clear answer to this second question. However, recent findings by Nagoshi demonstrate that cell division can indeed influence circadian period length [23], although it is not clear whether this effect on circadian period length is a gating effect on the circadian clock. Regarding the first question, the current opinion emphasizes the role of circadian clock in genome stability maintenance [24]. In order to obtain meaningful answers to these questions, one has to have a closer look at the molecular mechanisms of the circadian clock and the cell cycle engine. Because circadian rhythms involve complex transcriptional feedback loops, unperturbed transcriptional regulation of clock genes is critical for the stability of circadian rhythms. This was partially supported by the observation that treatment with the reversible transcription inhibitor 5,6-dichloro-1-beta-D-ribobenzimidazole alters both circadian phases and periods in the isolated eye of Aplysia [10]. During cell cycle progression, transcriptional regulation continuously changes. The most prominent changes occur at M-phase when the chromosomes condensed into compact structures. Most factors necessary for active gene expression are inaccessible to their binding site on DNA and cells undergo global transcriptional inhibition. In proliferating cells, this cell cycle-dependent transcriptional regulation occurs simultaneously with transcriptional programs of circadian regulatory machinery and, thus, transcriptional regulation events of these two molecular processes very possibly interact with each other. In this way, the two periodic molecular clock processes may interlock, especially during the global transcriptional inhibition during M-phase, which could potentially disturb the transcriptional feedback loops of the circadian clock machinery. With this possibility in mind, we reasoned that gating of the cell division cycle might help to minimize or eliminate potential disturbance of the transcriptional feedback loops of the circadian rhythm machinery.

It is not easy to experimentally study the cell cycle mediated effects of transcription inhibition on the circadian clock. It is, however, feasible to investigate this problem with mathematical modeling. A number of modeling approaches have already been successfully employed to individually study circadian clocks and the cell cycle [1], [25]–[28]. Modeling can not only reveal the underlying intrinsic molecular design principles of circadian clocks and the cell cycle machinery, but also help to predict and identify unknown components and regulatory principles. For example, using mathematical modeling approaches, Locke and colleagues predicted the presence of a new regulatory loop in the plant circadian clock system, which was supported by experimental results [29].

In this study, we investigate the hypothetical effects of global transcription inhibition in cell cycle M phase on the properties of the mammalian circadian clock and explore the implications of this effect on circadian gating of the cell cycle. Our simulation results show that transcriptional inhibition could entrain the circadian clock and at equilibrium entrainment, transcriptional inhibition pulses are always located at certain circadian phases, where they minimize inhibition induced circadian perturbation.

## Results

### Entrainment of Circadian Period by Transcriptional Inhibition at Constant Darkness Condition (DD Condition)

**Figure 1:**
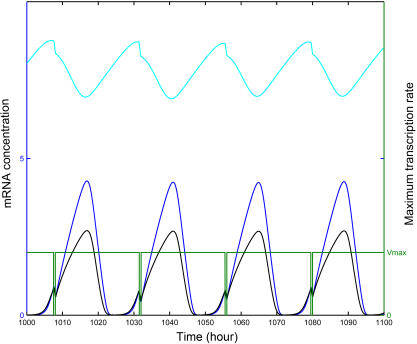
Square wave representing the transcriptional inhibition during cell cycle M phase. The square wave (green) oscillates between a maximum value, which represents the maximum transcription rate of the mRNAs, and zero, which represents the inhibition of transcription during M phase. The period of the square wave represents cell cycle period. The transcription of the three mRNA species (black, blue, and cyan) are simultaneously inhibited during the M phase.

**Figure 2:**
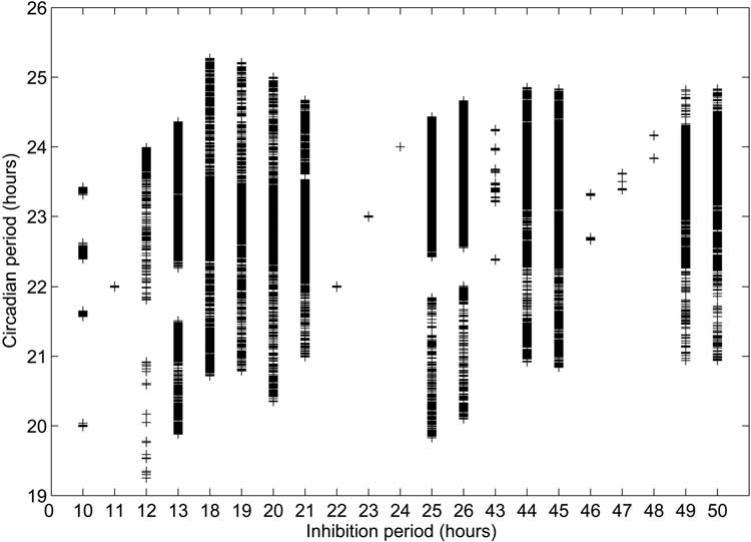
Effects of cell cycle M-phase on circadian periods. Cell cycle M-phase is introduced into the mammalian circadian model as transcriptional inhibition cycles of different periods. For each period, transcriptional inhibitions are imposed at various circadian phases with an interval of 30 minutes. The resulting models are simulated. Simulation data are sampled at equilibrium state and circadian periods are calculated for each simulation. The calculated periods are combined and plotted against transcriptional inhibition periods.

Entrainment of a circadian cycle to light is a well established biological observation. Light induced transcriptional alteration or protein degradation contributes to such entrainment. To assess whether M-phase transcriptional inhibition can also serve as an entrainment cue for the circadian clock, we numerically simulated a mammalian circadian model modified from the model published by Goldbeter et.al. [30] by incorporating periodic transcriptional inhibition (we will call this modified model henceforth the “coupled model”) using fourth and fifth order Runge-Kutta method. In the coupled model, the cell cycle M-phase was mimicked by periodic transcriptional inhibition of clock genes. With this modification, maximum transcription rates of clock genes fluctuate according to a square wave (Figure 1). The trough phase of the square wave represents M phase where transcription activities lower down to zero, while the peak phase represents other phases where transcriptions take place unchanged. The cycling period was set between 10 to 50 hours with steps of one hour, which practically covers the spectrum of mammalian cell cycle periods. Figure 2 gives an overview of the equilibrium circadian periods of the coupled system. When cells divide with a period close to 23.85 hours, which is the intrinsic period of the original mammalian circadian model from Goldbeter *et. al*., the equilibrium period of the coupled system is constant and equal to the imposed cell cycle period regardless of the circadian phase of the initiation of the M-phase transcriptional inhibition. This clearly indicates that entrainment occurs. Interestingly, such entrainment also occurred with a cell cycle period of 11 hours, approximately one half of 23.85 hours, or of about 48 hours (46, 47 and 48 hours in Figure 2), twice the 23.85-hour period. At other cell cycle periods, entrainment occurred irregularly and was strictly dependent on the phase of the circadian rhythm where transcriptional inhibition is initiated (data not shown). This latter case can be referred to as conditional entrainment. Although we did not extend our simulation to cycle periods longer than 50 or shorter than 11 hours, we think the extrapolation is reasonable.

**Figure 3:**
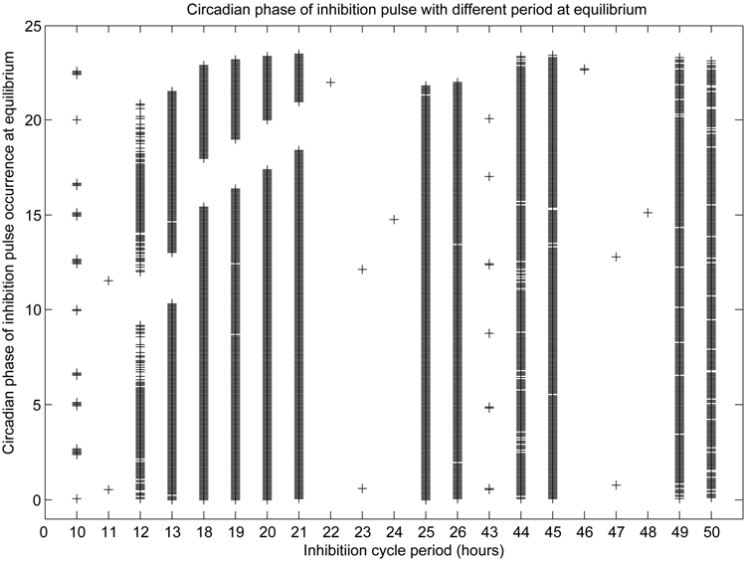
Steady state circadian phases of cell cycle M-phase. The cell cycle M-phase is introduced into the mammalian circadian model as transcriptional inhibition cycles of different periods. For each period, transcriptional inhibitions are imposed at various circadian phases with an interval of 30 minutes. The resulting models are simulated. Simulation data are sampled at equilibrium state and the circadian phases where cell cycle M-phase is located are calculated for each simulation. The calculated phases are combined and plotted against transcriptional inhibition periods.

Next, we assessed the distribution of cell cycle M-phase (transcriptional inhibition pulse) on the circadian phase of the coupled system at equilibrium entrainment. To this end, the phases of the circadian cycles where inhibition pulses occurred were determined at equilibrium of every simulation and plotted against the cell cycle periods. As shown in Figure 3, patterns similar to those in Figure 2 emerge. At cell cycle periods close to half of 24 h, 24 h or twice 24 h, where period entrainment occurs, inhibition pulses were also entrained to specific circadian phases. At other phases of the period, no such phase entrainment could be detected.

**Figure 4:**
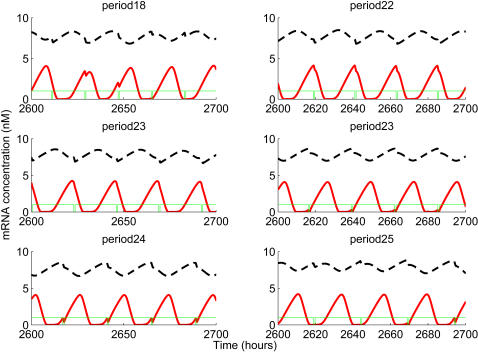
Steady state circadian phases of cell cycle M-phase for periods of 22, 23 and 24 hours. Simulations are performed as described in Figure 1 and Figure 2. Dynamics of different state variables are directly plotted.

Figure 4 shows the details of the simulation results for cell cycle periods of 18, 22, 23, 24 and 25 hours, where entrainment occurred at periods of 22, 23 and 24 hours. For the 22 hours cell cycle period, the circadian cycle period was strictly entrained to 22 hours. The standard deviations of the circadian periods were for none of the circadian phases larger than 0.1 h (data not shown). The inhibition pulse occurred at a single circadian phase close to peak of Per mRNA curve which is defined as CT0. Similar strict entrainment was also observed at a period of 24 hours. In this case, the circadian period was entrained to 24 hours and the inhibition pulse occurred at a single circadian phase close to CT13. There is a subtle difference between the case of a 23 h period and the 22 and 24 h periods. The circadian cycle of the 23 h period was still entrained to 23 hours, but equilibrium inhibition pulses occurred at two circadian phases, one that was close to CT0 and another close to CT13, corresponding to the entrainment phases of the 22 and 24 hour periods, respectively.

### Clock Gene mRNA Synthesis Rate Curves

**Figure 5:**
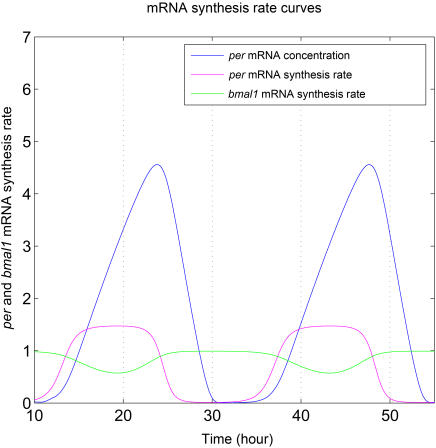
Per and Bmal1 mRNA synthesis rate curves.

If inhibition occurs at circadian phases where synthesis of clock gene mRNAs are actively expressed, circadian rhythms will possibly be perturbed. However, if inhibition occurs at circadian phases either without clock gene mRNA expression or with balanced synthesis of two antagonistic genes, there will be no or minimal effect on the circadian clock. Figure 5 displays the mRNA synthesis rates of clock genes across the circadian period. Since the synthesis of Per mRNAs (NM_011065.3, NM_011066.3, NM_011067.2) and Cry mRNAs (NM_007771.3, NM_009963.3) are roughly in-phase, only the synthesis rates of Per mRNA and Bmal mRNA (NM_007489.3) are displayed in Figure 5. The synthesis rate curves of the two mRNA molecules intersect at two points across the circadian period. These two intersection points are close to those two locking circadian phases where inhibition pulses occurred at equilibrium, as shown in Figure 4. Since the syntheses of the Per and Bmal1 mRNAs oscillate in anti-phase, transcriptional inhibition at any point other than these two intersection points will lead to unbalanced inhibition, e.g. the less the inhibition of one gene, the greater that of the other, thus resulting in larger system perturbations. On the other hand, inhibition at these two points results in equal inhibition of both molecules and thus the least perturbation of the circadian clock. This would explain why entrainment of the circadian clock by the cell division cycle always occurs at these two phase points.

### Differences between Transcriptional Inhibition Induced Perturbations at Different Phases of a Light–Dark Cycle (LD Condition)

**Figure 6:**
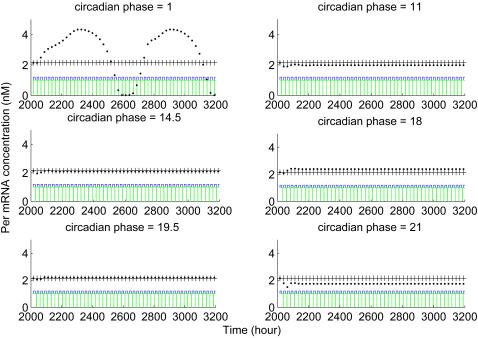
Transcriptional inhibition induced changes at different circadian phases under LD cycle conditions. The LD cycle is first introduced into the circadian model, and the resulting model is simulated. When the model reaches equilibrium, transcriptional inhibitions are introduced into the model at different circadian phases. The system changes after inhibition imposition is depicted by the difference in Per mRNA level at light onset between pre- and post-inhibition imposition. “+” denotes Per mRNA level at light onset before inhibition imposition; “.” denotes Per mRNA level at light onset after inhibition perturbation.

Our simulation so far studied the effect of M-phase transcriptional inhibition in DD condition. In reality, light cycle and cell cycle always influence the circadian cycle simultaneously. Furthermore, experiments studying circadian entrainment of cell cycle phases are all conducted under the condition of a light-dark cycle. To directly compare experimental results with our simulation, we have to introduce a LD cycle into our model. Our working hypothesis is that entrainment of cell cycle phases, especially of the M-phase, to certain circadian phases is meant to minimize circadian perturbation induced by cell cycle progression, in particular by M-phase global transcriptional inhibition. Our objective is to determine whether, in the presence of a LD cycle, one or more circadian phase(s) can be identified, where the imposition of transient transcriptional inhibition does not significantly alter the circadian cycle. To this end, we conducted simulations with a model incorporating both a light-dark cycle and transcriptional inhibition cycle effects. There are three ways to conduct such a simulation study. Two different effects can be introduced either simultaneously or sequentially. Since mammals normally live under light-dark cycle conditions, we assume a light cycle factor intrinsic to the mammalian circadian clock and that a LD cycle is the background condition of other molecular processes. Thus, we first introduced a light cycle into the model, and the transcriptional inhibition cycle was introduced after the system reached a new equilibrium state. Since human and mouse cells *in vivo* normally show proliferation with a periodicity of 24 h or longer, we began with a 24 h transcriptional inhibition cycle. The results show that, as under the DD condition, the transcription inhibition cycle altered phase and period of the circadian clock. The magnitude of change depends on the phase of the circadian cycle at which transcriptional inhibition is imposed. Transcriptional inhibition initiated at some circadian phases induced large changes of the system, which took a long time to relax into a new equilibrium state. In these cases, systems normally do not return to the previous equilibrium state. On the other hand, imposing transcriptional inhibition at certain other circadian phases induced relatively small changes of the system, which rapidly returned to the previous equilibrium. At still other circadian phases, transcriptional inhibition induced no system changes at all. Some aspects of our results are shown in Figure 6. It is apparent that at a circadian phase close to 14.5 and 19.5 (phase 0 corresponds to onset of light, CT0), little perturbation was induced by transcriptional inhibition (middle and bottom panels of left Figure 6), while at other phases, larger deviations were observed (right side Figure 6). At phase 1, the system simply transits into quasi-periodicity (top panel of left Figure 6)When simulations were performed with transcriptional inhibition cycles of periods other than 24 hours, phases where transcriptional inhibition induced minimum or no changes can not be detected.

We further did similar simulation study in the mammalian circadian model with 19 equations published by Goldbeter et al. [30] and a Drosophila circadian model published by Udea et al. [31] to see whether this kind of phase specific difference also exists in other circadian models. Our results clearly indicated that these different models also exhibit this phase specific difference in transcriptional inhibition induced perturbation although the exact phases where transcriptional inhibition induced lest perturbations in Drosophila model are different from the two mammalian models (see Figure S1 and Figure S2).

### Noise Has Little Effect on the Entrainment of the Circadian Clock by Cell Division

**Figure 7:**
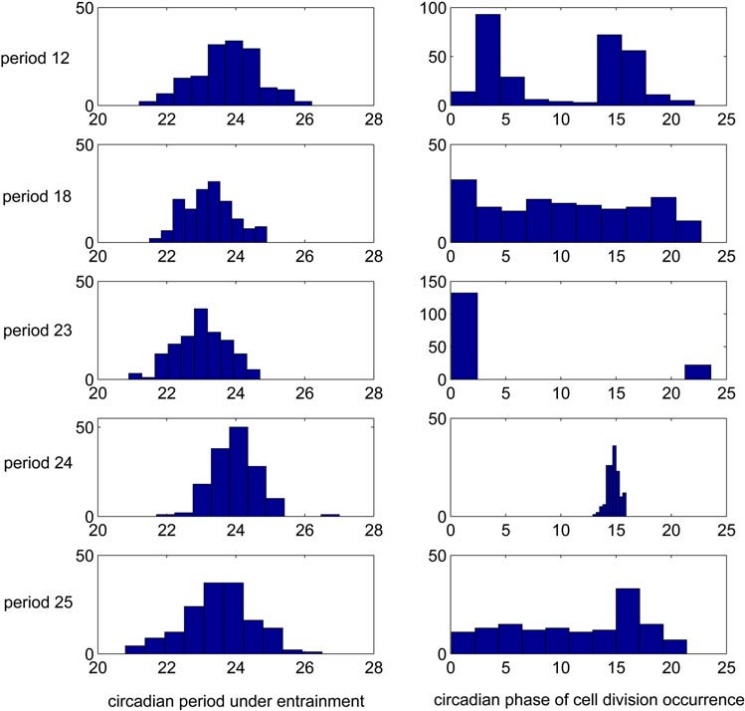
Entrainment of circadian clock by cell cycles of different periods at constant darkness in the presence of molecular noise. The effects of the cell cycle period on the entrainment in the presence of noise were studied by changing the periods of the square waves imposed onto the circadian model with noise. The periods of the coupled model and the circadian phases (with the peak of Per mRNA as the reference phase) where the troughs of the square wave occurred are determined. The distributions of the resulting circadian periods and the phases of transcriptional inhibition occurrence resulting from one simulation are displayed here.

**Figure 8:**
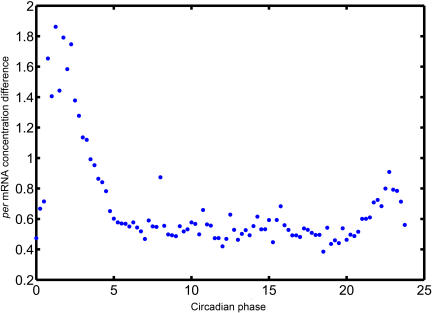
Phase specific differences in cell division induced circadian system alterations in the presence of noise. Light dark cycles and transcriptional inhibition cycles initiated at different circadian phases (with light onset as the reference) were imposed onto circadian models, and the Per mRNA concentration differences at light onset before and after transcriptional inhibition were calculated. Results from 100 simulations were averaged.

It has been demonstrated that circadian systems are robust to molecular noise and entrainment of circadian clock by light cycles can occur in the presence of molecular noise [32],[33]. To study the effect of noise on the entrainment of circadian clock by transcriptional inhibition cycles, noises were introduced into the differential equations of the mammalian circadian model. System trajectories of the model were then simulated as above mentioned. Simulation results showed that the model exhibits robust periodic behavior in the presence of noise (see Figure S3) and such periodic behavior remained when either light cycles or transcriptional inhibition cycles is imposed onto the model (data not shown). For transcriptional inhibition cycles, those with periods close to 24 hours are easier to entrain the model, reflected by more focused distribution of the circadian phases where inhibition pulses occur and more centered distributions of entrained circadian periods to values identical to transcription inhibition cycle (Figure 7). When transcriptional inhibition cycles and light cycles of 24 hour are imposed onto the model, inhibition cycles fluctuating with specific phasing relationships with light cycles will induce lest rhythms changes in the model system (Figure 8). These results are compatible with the previous results in the absence of noise.

## Discussion

Interactions between the circadian clock and the cell cycle engine have been suggested by many experimental observations in various organisms [11], [15], [20], [34]–[41]. However, the interaction and communication structure between these two systems remain to be revealed. In this study, we applied a computational simulation approach to this problem. Our results show that global transcriptional inhibition during the cell cycle M phase can shift the circadian phase and serve as entrainment cue for the circadian clock.

Experimental observations suggesting an interaction between the circadian clock and the cell cycle are, in most cases, simply the non-random distribution of certain cell cycle events across circadian phases or fluctuations of cell cycle regulatory gene expression with circadian periodicity. Mechanistic details of this interaction are so far not known, yet in some instances, specific molecular links have been proposed [35],[42]. In 2003, Matsuo et al. provided the first evidence in mouse that Wee1, an important cell cycle regulator kinase, is under direct control of circadian clock genes and that both Wee1 expression and mitosis follow a circadian rhythm. This report provides support for the idea that the circadian clock must have a direct influence on cell cycle progression. Based on this assumption, Calzone *et al*. created a coupled model of circadian clock and cell cycle (https://hal.ccsd.cnrs.fr/docs/00/07/01/91/PDF/RR-5835.pdf). Since a potential influence of the cell cycle on circadian clock was not considered in their coupled model, it exhibited a bias towards the effects of the circadian clock on the cell cycle, while any reverse effect was neglected.

To simulate the effects of the cell cycle on the circadian clock, appropriate molecular links have to be identified and corresponding parameters have to be determined. Compared to the evidence for a dependence of the cell cycle on the circadian clock, evidence for the reverse effect is rare. The most pertinent evidence came from fluorescent imaging of gene expression in individual NIH3T3 mouse fibroblasts with circadian rhythm [23]. It was found that cell division shifted the period length of the circadian clock. Although there is no direct evidence of the molecular mechanism underlying this phenomenon, the period length change after cell division was attributed to global transcription inhibition during cell division. Interestingly, transient transcriptional inhibition by chemicals has been demonstrated by Eskin et al. to be able to alter circadian phases and periods [10]. Considering these observations and the fact that the most prominent transcriptional change during cell cycle progression is global transcriptional inhibition associated with cell division, it is reasonable to assume that cell cycle events, in particular cell division at M-phase, exert direct effects on circadian clock.

We thus focused here on the potential effects of M-phase global transcriptional inhibition on the circadian clock. One has to bear in mind, however, that cell cycle progression involves complicated transcriptional, translational and post-translational regulations. Consistent with Eskin's experimental observation, our simulation study confirmed that transcriptional inhibition changed both phase and period of the circadian clock.

Two interesting points emerge from our computational simulation. The first one is the entrainment of the circadian period by the cell cycle. This entrainment occurs only at cell cycle periods close to one half, twice or equal to the intrinsic circadian model period of 23.85 h, namely 11, 22, 23, 24, 46, 47 and 48 h. At other cell cycle periods, entrainment rarely occurred. The second point is that when the circadian clock system reaches a new equilibrium state after perturbation by periodic transcriptional inhibition, the circadian phase(s) where transcriptional inhibition pulses are locked, is (are) focused rather than randomly distributed across the whole circadian clock period. For the 22 hour period, transcriptional inhibition remains at the circadian phase following the Per mRNA peak, for the 23 hour period, two steady state phases exist, one equivalent to that of the 22 hour period, the other one close to the middle between two Per mRNA peaks. For the 24-hour period, one unique steady state appears again, in this case close to the middle between two Per mRNA peaks.

Further inspection showed that these positions are close to phases where the synthesis rate curves of the Per and Bmal1 mRNAs intersect. It is evident that at the intersection points, the difference between the synthesis rates of these two molecules is zero and transcriptional inhibition pulses influence their synthesis to the same extent. According to the accepted mechanism of circadian clock regulation, Per exerts a negative feedback on itself, but positively affects Bmal1 expression. Similarly, Bmal1 regulates itself negatively, but regulates Per positively. This regulation regime causes an anti-phasic oscillation of these two molecules with respect to each other. When transcriptional inhibition is imposed on the circadian system, several different responses occur, depending on the circadian phase where transcriptional inhibition happens. At circadian phases where Bmal1 synthesis rate reaches maximum and Per synthesis rate is zero, transcriptional inhibition induces maximum delay of accumulation of Bmal1 mRNA, but does not affect Per mRNA synthesis. At these circadian phases, transcriptional inhibition causes maximal perturbation of the circadian system. At other phases, transcriptional inhibition delays the accumulation of one of these two mRNAs, while accumulation of the other is accelerated. The effects are also quantitatively different, depending on the exact circadian phase of transcriptional inhibition. In some phases, transcriptional inhibition delays Per mRNA accumulation but accelerates Bmal1 mRNA accumulation, while in other phases the reverse is observed. The influence on one mRNA is always associated by a simultaneous influence on the other mRNA. The magnitude of counterbalance is determined by the difference between the synthesis rates of the two molecules at that phase. The more the disturbances are balanced, the less is the circadian system affected by the transcriptional inhibition at that circadian phase. It is obvious that near the intersection points of Figure 5, the influences are more balanced than at all other points and thus, the circadian system is less perturbed by transcriptional inhibition at phases near those points. For stable entrainment of the circadian clock, two conditions must be satisfied. One is that the circadian system must not be drastically perturbed. The other one is that the phase shift induced by the entraining cue equals the difference between the unperturbed period and the entraining cycle period. At phases near the intersection points, transcriptional inhibition induced perturbations and phase shifts satisfy these two conditions for steady entrainment, while at other phases they are less likely to be met. We assume that these special characteristics of phases near intersections may explain the fact that in most cases of the steady entrainment of the circadian clock by periodic transcriptional inhibition, inhibition pulses were, without exception locked at these unique circadian phases.

Still, at cell cycle periods other than those mentioned above, transcriptional inhibition pulses were also found locked to other phases, e.g. circadian phase distribution for 10 and 43 hours in Figure 3. We cannot yet explain this complex pattern. Further work has to be undertaken to unravel this complexity. In mouse fibroblasts cultures, it was found that cell division mainly occurred at three phases with an interval of roughly 8 hours. The reason for this discrepancy between observations in fibroblasts and our simulation is not clear. It may reflect differences between the endogenous fibroblasts circadian clock and the circadian model we used and/or differences between *in vitro* and *in vivo* conditions.

In the physiological context, a circadian clock is always under the influence of a light-dark cycle. To place our simulation in a more physiological context, we also simulated the cell cycle and circadian clock interaction in the presence of a light-dark cycle. To this end, we incorporated both a light-dark cycle and the transcriptional inhibition cycle into the mammalian model. Our simulation results revealed two windows in the circadian cycle, where transient transcriptional inhibition induced only transient and small alterations to the circadian clock regulatory system. With the beginning of the light cycle taken as the 0 reference phase (CT0), one window is close to 15 h, and the other window is close to 19 h, corresponding to the middle and late night respectively. Although there is to our knowledge no experimental evidence for mammals supporting the entrainment of cell cycle M-phase to circadian phases close to the first window in our simulation, evidence from a mouse liver regeneration study revealed indeed the entrainment of hepatocyte cell cycle mitosis to phases close to this second window [42]. There are also reports on a circadian rhythm of the cell cycle M-phase in mouse and human skin and mouth mucosa epithelia [40],[43]. According to one of these studies, mitosis occurs mainly at a phase roughly corresponding to the time before sunset [40]. This is in contrast to proliferating hepatocytes and the results of our simulation. Considering that cells of different tissue origin display distinct physiological circadian rhythms, the differences in occurrence of cell cycle M-phase between skin and mucosa epithelia and hepatocytes and our simulation study are not surprising. We do similar simulations with the mammalian circadian model of 19 equations from Goldbeter et.al. The results are similar to those of the 16 equation model. More interestingly, simulations with a Drosophila circadian model also revealed the existence of minimum perturbation at certain circadian phases. This indicates that circadian phase specific minimum perturbation by transcriptional inhibition is general to circadian systems from different species. The partial overlap between the simulated circadian phases with the smallest impact of transcriptional inhibition on the circadian clock and those experimentally observed circadian phases where mitosis most frequently occurs, suggests that the principle of minimal circadian perturbation might, at least partially, contribute to the phenomena of circadian entrainment of cell cycle mitosis in mammals. We also performed simulations with transcription cycle periods other than 24 hours. In these cases, steady entrainment can not be detected. This clearly means that cell cycles with periods different from circadian period can not result in steady entrainment and have to be gated by circadian clock to obtain steady coupling between circadian clock and cell cycle.

The current view of circadian entrainment of the cell cycle is that the circadian clock helps to maintain genome stability by timing mutation sensitive cell cycle phases to circadian phases with least exposure to mutagens. Our simulation suggests that circadian entrainment of the cell cycle could also help to maintain circadian clock stability by minimizing cell division induced perturbation of the circadian clock. These two notions are not mutual exclusive. They complement each other and in combination provide for a fuller picture of an elusive phenomenon.

In summary, highly regulated transcriptional processes are critical for normal functioning of the circadian clock. Global transcriptional inhibition during M-phase of the cell cycle might perturb normal progression of the circadian clock, and there might be circadian windows where transcriptional inhibition has little influence on normal circadian progression. One could therefore expect to find (a) molecular mechanism(s) which places the M-phase of the cell cycle in such windows to minimize or eliminate cell cycle induced perturbation. Our study is the first attempt to tackle this problem by computational simulation, and our results support this hypothesis.

## Materials and Methods

### Circadian Model

The circadian model used in this study is from the mammalian model published by Leloup and Goldbeter in 1993 [30]. There are two versions of this model. One version is composed of 16 differential equations and, the other one is composed of the same 16 equations plus three additional equations. The 16 shared equations describe the dynamics of the Per, Cry, and Bmal1 mRNAs and their corresponding proteins. The additional 3 equations in model 2 describe the dynamics of the Rev-erbalpha mRNA (NM_145434.3) and proteins. The two models gave similar simulation results. These models reflects mRNA transcriptional regulation, protein phosphorylation regulation and protein compartmental transportation dynamics (see Figure S4 for details). The dynamic behaviors of these models are generally in agreement with characteristic features of mammalian circadian clocks. For details of the equations and descriptions, we refer the readers to the original publication by Leloup and Goldbeter [30]. A Matlab ODE file for the modified model is also provided (see Text S1).

### Incorporation of the Effect of M-Phase Transcriptional Inhibition into the Circadian Model

We did most of our simulations with the 16 equation model. In Goldbeter's circadian model, the dynamics of three clock gene mRNA levels are governed by the following three equations:
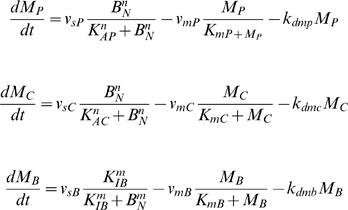
where M_P_, M_C_, M_B_ denote the Per, Cry and Bmal1 mRNA, respectively. v_sP_, v_sC_, v_sB _represent the maximum transcription rates of the Per, Cry and Bmal1 mRNA, respectively.

To incorporate the effects of cell cycle M-phase global transcriptional inhibition on the circadian clock, we modified Leloup's mammalian circadian model by letting parameters v_sP_, v_sC_, v_sB_ oscillate between the optimized values of the original model and zero (or other values below optimum). The oscillation of these parameters reflects the periodic cell cycle M-phase. The periods of oscillation of these parameters mimic the cell cycle period, and the differences between the two oscillating values reflect the degree of M-phase transcriptional inhibition.

Although it is well known that chromosomes are highly condensed and transcription is globally inhibited during M-phase, there is no quantitative experimental result concerning the duration and extent of transcription inhibition in M-phase. Because the M-phase of the mammalian cell cycle lasts roughly 1–2 hours and is relatively constant compared to other cell cycle phases, we assume that the variation of these three parameters follows a square wave with a trough phase of relatively constant length of 30 minutes corresponding to the M-phase transcriptional inhibition pulse. We assume that transcription inhibition of circadian clock genes occurs at least at the middle part of M-phase. Based on this assumption, a duration of 30–60 minutes (roughly half the mammalian cell cycle M-phase length) of transcription inhibition is introduced into the model.

To implement this modification, we introduced a new parameter v into the original model, whose value is governed by the following formula:
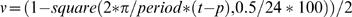
in which *square* is a square wave function, *period* denotes period of transcriptional inhibition, representing cell cycle period, *t* denotes time and *p* denotes the circadian phase with which we can control where the inhibition pulse begins.

To simulate oscillation of Per, Cry and Bmal1 mRNAs, v_sP_, v_sC_ and v_sB_ are all multiplied with the parameter v. The three equations governing the dynamics of the three mRNAs are thus modified as follows:
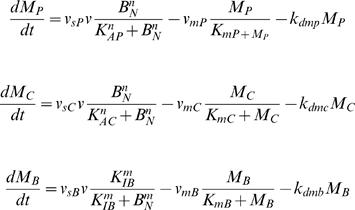



In this way, the decline of v_sP_, v_sC_ and v_sB_ mimics transcriptional inhibition, and the period of variation reflects the cell cycle period. We treat the two terms of transcriptional inhibition and cell cycle M-phase global inhibition as interchangeable in this study.

### Introduction of Noise into the Circadian Model

To study the effect of noise on the entrainment properties of periodic transcriptional inhibition, we introduced a white noise term into the differential equations of the original model as follows:
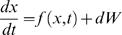
where *dW* = *δ* * *G*, with *δ* controlling the magnitude of the noise and *G*representing the Gaussian process. Noise terms were added into one or several different equations to find a proper way to introduce noise into the model. In this study, we just add a noise term into the third equation governing the dynamics of Bmal1 mRNA concentration, which functions as an important regulatory factor for circadian clock. The equation with noise term is as follows:
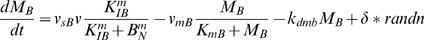



### Lists of Genes and Proteins Included in the Mammalian Circadian Models

Although the mammalian circadian models we used in this study reflect general properties of mammalian circadian clock, the parameters are basically estimated from data collected from mouse experiments. So we just list mouse Refseq accession numbers for the genes and proteins. The three Per genes and proteins are collectively represented as one Per gene and protein respectively in the model and the two Cry genes and proteins are treated as is.

Genes: *Clock* (NM_007715.5); *Per1* (NM_011065.3); *Per2* (NM_011066.3); *Per3* (NM_011067.2); *Cry1* (NM_007771.3); *Cry2* (NM_009963.3); *Bmal1* (NM_007489.3); *Rev-ERBa* (NM_145434.3). Proteins: CLOCK (NP_031741.1); PER1 (NP_035195.1); PER2 (NP_035196.2); PER3 (NP_035197.2); CRY1 (NP_031797.1); CRY2 (NP_034093.1); BMAL1 (NP_031515.1); REV-ERBA (NP_663409.2).

## Supporting Information

Figure S1 Transcriptional inhibition induced changes under LD cycle conditions in the Goldbeter mammalian circadian model with 19 equations. The LD cycle is first introduced into the circadian model, and the resulting model is simulated. When the model reaches equilibrium, transcriptional inhibition is then introduced into the model. The system changes after inhibition imposition is depicted by the difference in Per mRNA level at light onset between pre- and post-inhibition imposition. “+” denotes Per mRNA level at light onset before inhibition imposition; “.” denotes that Per mRNA level at light onset after inhibition perturbation.
(0.45 MB TIF)

Figure S2 Transcriptional inhibition induced changes under LD cycle conditions in the Udea Drosophila circadian model. Methods and interpretations are the same as Figure S1.
(0.51 MB TIF)

Figure S3 Circadian oscillations are robust to noise. Noises are introduced into the mammalian circadian model as described in the Materials and Methods section. The magnitude of the noise is controlled by σ .
(0.83 MB TIF)

Figure S4 Molecular processes included in the mammalian circadian models we used in this study (adapted from [30]). Ovals represent proteins and rectangulars represent mRNA transcription. Black elements denote protein degradation. cyto(-) and nuc(-) represents cytoplasmic and nuclear proteins respectively. -P denotes protein phosphorylation. Lines with arrows means protein phosphorylation and dephosphorylation activation or transcriptional activation, while lines with bars means inhibition. The green colored molecules at the upper-left corner are only included in the 19 equation models, while the light blue colored molecules are included in both mammalian models.
(0.42 MB TIF)

Text S1 ODE file for the mammalian circadian model incorporating transcriptional inhibition.
(0.01 MB TXT)
