# Correction: Modeling the Effects of Cell Cycle M-phase Transcriptional Inhibition on Circadian Oscillation

**DOI:** 10.1371/annotation/3cf61cf0-5b5d-4550-adc8-42580b7a3540

**Published:** 2008-07-04

**Authors:** Bin Kang, Yuan-Yuan Li, Xiao Chang, Lei Liu, Yi-Xue Li

Author affiliation 1 is incorrect. The correct affiliation is: Key Laboratory of Systems Biology, Shanghai Institutes for Biological Sciences, Chinese Academy of Sciences, Shanghai, China

