# Supplementary figures and images for: Modeling the Effects of Cell Cycle M-phase Transcriptional Inhibition on Circadian Oscillation
Source: PLoS Comput Biol. 2008 Mar 28;4(3):e1000019. doi: 10.1371/journal.pcbi.1000019 (PMC2267494; doi:10.1371/journal.pcbi.1000019)

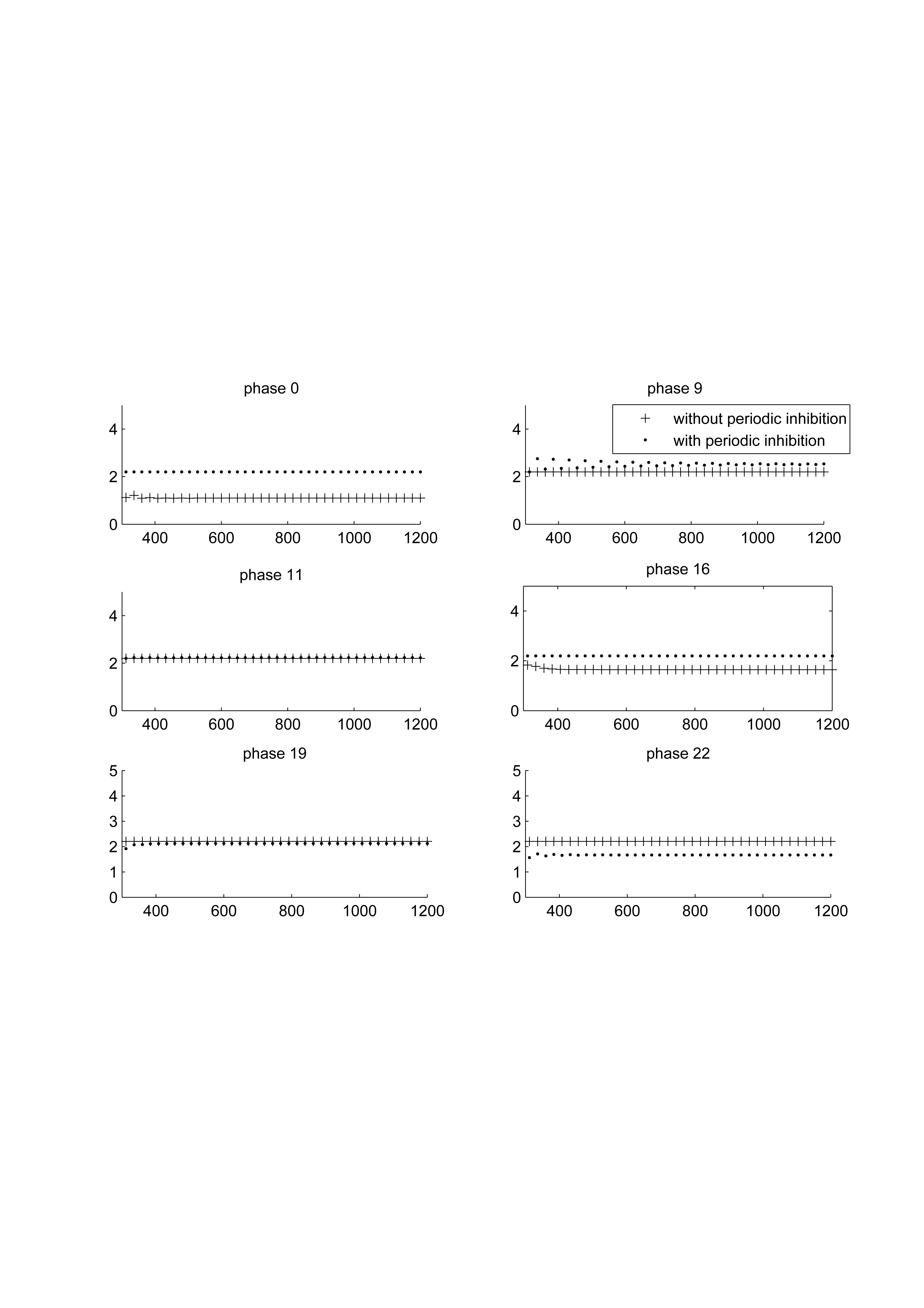

Supplement: Figure S1 — Transcriptional inhibition induced changes under LD cycle conditions in the Goldbeter mammalian circadian model with 19 equations. The LD cycle is first introduced into the circadian model, and the resulting model is simulated. When the model reaches equilibrium, transcriptional inhibition is then introduced into the model. The system changes after inhibition imposition is depicted by the difference in Per mRNA level at light onset between pre- and post-inhibition imposition. “+” denotes Per mRNA level at light onset before inhibition imposition; “.” denotes that Per mRNA level at light onset after inhibition perturbation. (0.45 MB TIF) [file pcbi.1000019.s001.tif]

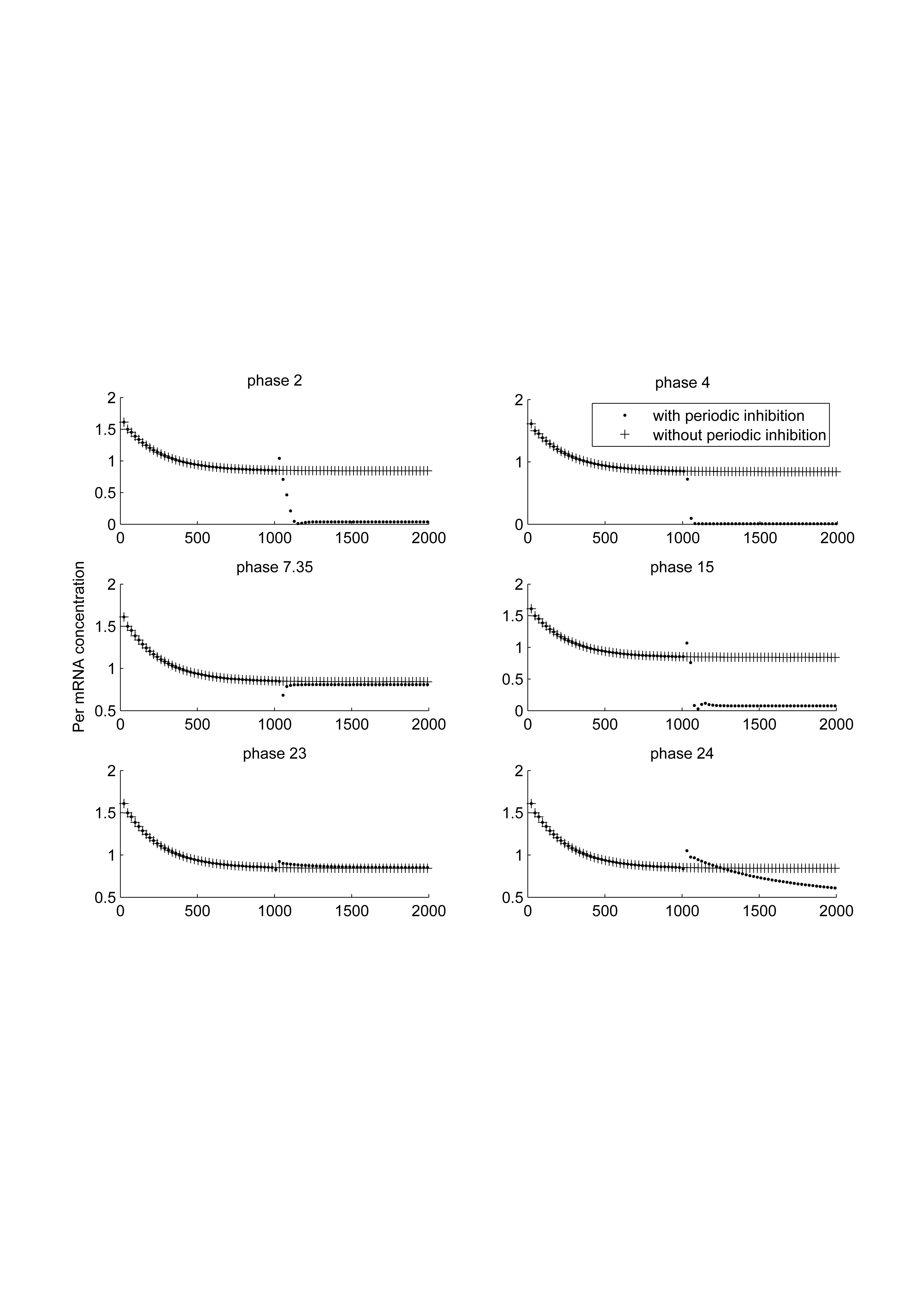

Supplement: Figure S2 — Transcriptional inhibition induced changes under LD cycle conditions in the Udea Drosophila circadian model. Methods and interpretations are the same as Figure S1. (0.51 MB TIF) [file pcbi.1000019.s002.tif]

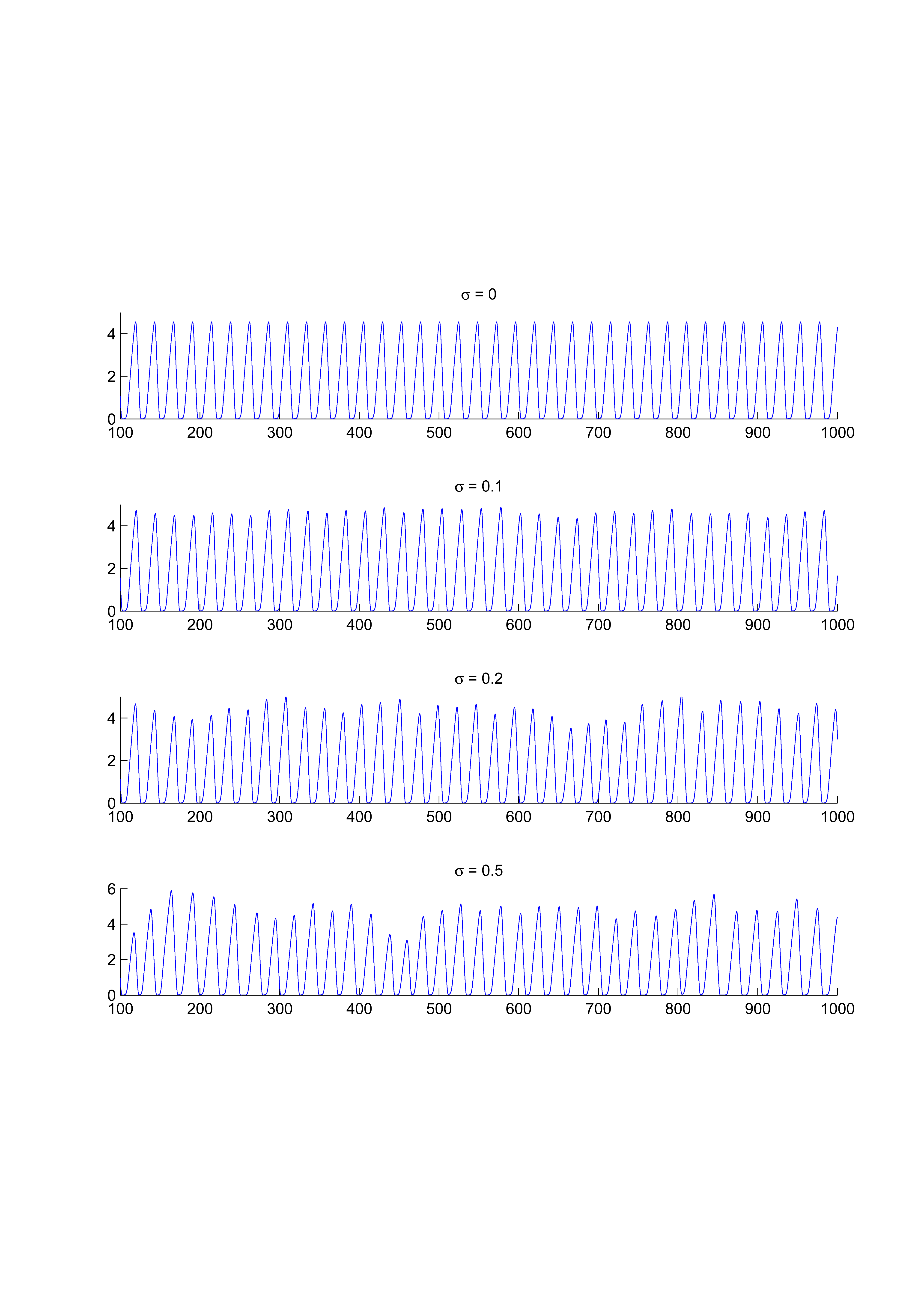

Supplement: Figure S3 — Circadian oscillations are robust to noise. Noises are introduced into the mammalian circadian model as described in the Materials and Methods section. The magnitude of the noise is controlled by σ . (0.83 MB TIF) [file pcbi.1000019.s003.tif]

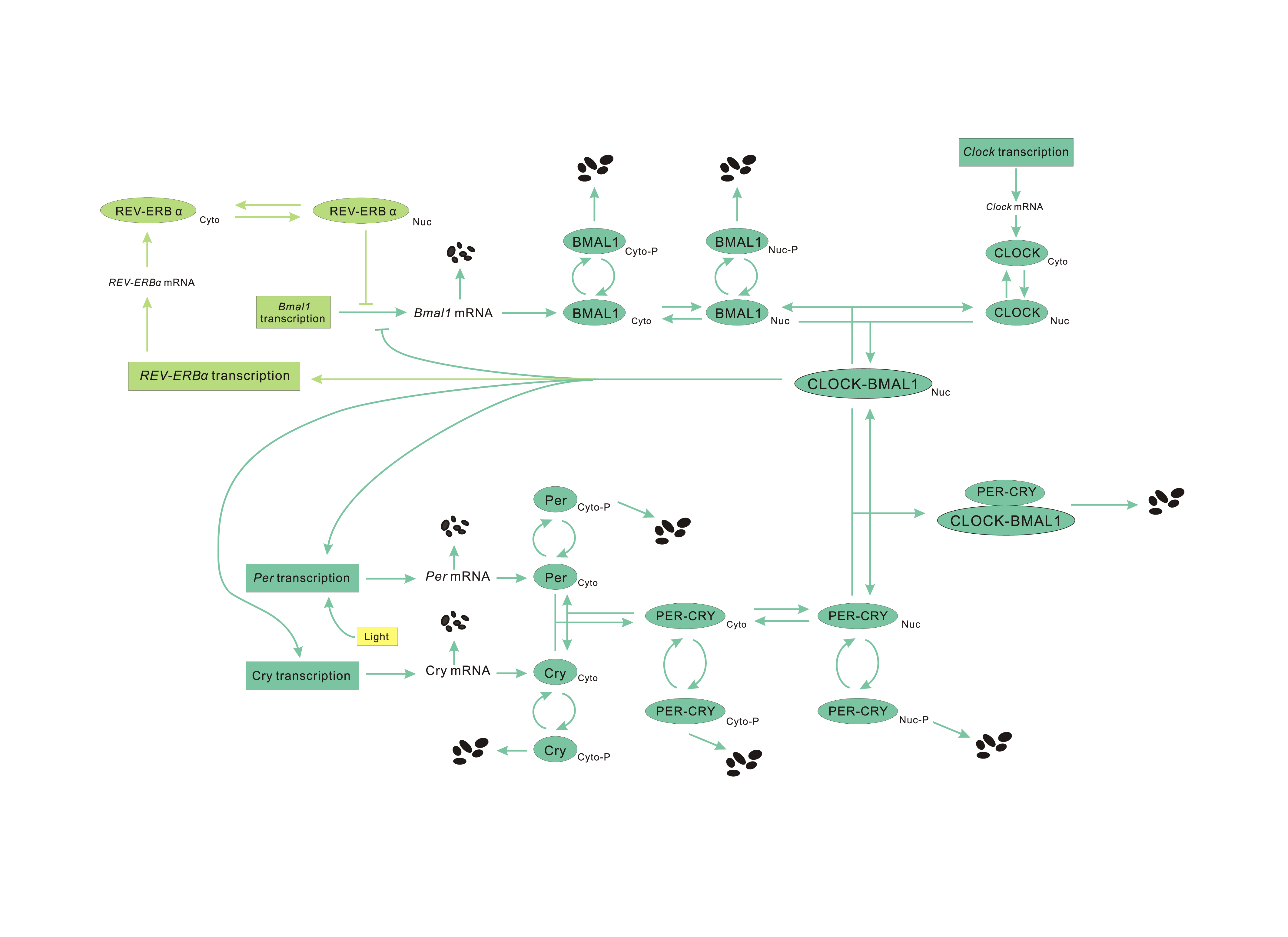

Supplement: Figure S4 — Molecular processes included in the mammalian circadian models we used in this study (adapted from [30]). Ovals represent proteins and rectangulars represent mRNA transcription. Black elements denote protein degradation. cyto(-) and nuc(-) represents cytoplasmic and nuclear proteins respectively. -P denotes protein phosphorylation. Lines with arrows means protein phosphorylation and dephosphorylation activation or transcriptional activation, while lines with bars means inhibition. The green colored molecules at the upper-left corner are only included in the 19 equation models, while the light blue colored molecules are included in both mammalian models. (0.42 MB TIF) [file pcbi.1000019.s004.tif]
